# A Case of Multivessel Coronary Artery Disease and Anomalous Origin of the Right Coronary Artery With a Malignant Course Presenting With Non-exertional Chest Discomfort and Brugada Phenocopy

**DOI:** 10.7759/cureus.33718

**Published:** 2023-01-12

**Authors:** Maan Kathryn L Gozun, Isaac Mizrahi, Mohammed Ali, Dipanjan Banerjee, Peter Tsai

**Affiliations:** 1 Internal Medicine, University of Hawaii, Honolulu, USA; 2 Cardiology, University of Hawaii, Honolulu, USA; 3 Cardiology, Queen's Medical Center, Honolulu, USA; 4 Cardiothoracic Surgery, Queen's Medical Center, Honolulu, USA

**Keywords:** tte: trans-thoracic echocardiography, svg: saphenous vein graft, scd: sudden cardiac death, rca: right coronary artery, pft: pulmonary function test, lima: left internal mammary artery, lad: left anterior descending, cto: chronic total occlusion, cabg: coronary artery bypass graft, caa: coronary artery anomaly

## Abstract

Coronary artery anomalies (CAAs) are an uncommon cause of chest pain in the younger population. Misdiagnosis can be detrimental and lead to sudden cardiac deaths. We present a 62-year-old male with a past medical history significant for chest pain history with a workup in 2001 presumed to be non-cardiac in origin from bronchial asthma. He presented from a Micronesian Island for the evaluation of non-exertional chest discomfort. Further workup showed a Brugada type I pattern on ECG and ST wave depressions on anterolateral and inferior leads with associated AVR elevation on exercise stress testing. Further ischemic workup with coronary angiography revealed right dominant circulation with three-vessel coronary artery disease (CAD), including mid-left anterior descending (LAD) artery chronic total occlusion (CTO) with the right to left collaterals, left circumflex, and right coronary artery (RCA) with the accompanied anomalous origin of RCA. The patient underwent surgical correction of the anomalous RCA and coronary artery bypass grafting for the multi-vessel CAD. CAAs are usually found incidentally during ischemic workups similar to this case. Patients with CAAs can be managed conservatively with caution regarding physical activity. However, high-risk patients will warrant surgical treatment to avoid sudden cardiac death. The diagnosis of CAAs can be challenging and prone to misdiagnosis and maltreatment. It may be beneficial to pursue this in younger patients with ischemia-like symptoms. Further studies should be performed to identify the true incidence and guide medical practitioners regarding the risks, costs, and benefits of diagnosing and surgically treating CAAs in the general population.

## Introduction

Coronary artery anomalies (CAAs) generally have an estimated incidence of 0.6-1.3% in angiographic studies [1-2]. However, a more recent study has demonstrated a higher incidence of 2.5% using a contrast-enhanced, multidetector computed tomography (CT) that was partially attributed to a study cohort with pre-selection of patients referred for coronary anomaly and loose definition, including more variants [3]. This incidence may even be underestimated, as a study performed in St. Luke’s Episcopal Hospital revealed an incidence of 5.6% [4]. CAAs include congenital conditions defined as any pattern of the three main epicardial coronary arteries rarely encountered in the general population affecting features such as origin, course, and termination [4,5]. We present a case of a 62-year-old male who came in for non-exertional chest discomfort presenting with Brugada type I pattern and was found to have multi-vessel coronary artery disease (CAD) and anomalous origin of right coronary artery (RCA) with a malignant course. This case tackles an anomalous origin of the right coronary artery (RCA) with a malignant course, which is an uncommon differential diagnosis for chest pain in the younger population. Misdiagnosis can be detrimental and lead to sudden cardiac deaths. High-risk features should be identified to justify definitive surgical management.

## Case presentation

**Figure 1:**
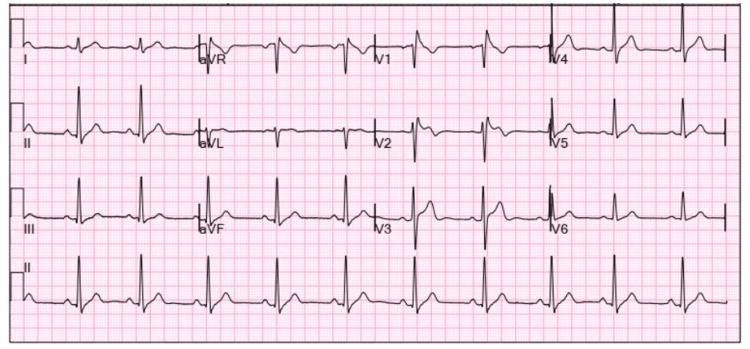
EKG strip demonstrating a Brugada type I pattern

**Figure 2:**
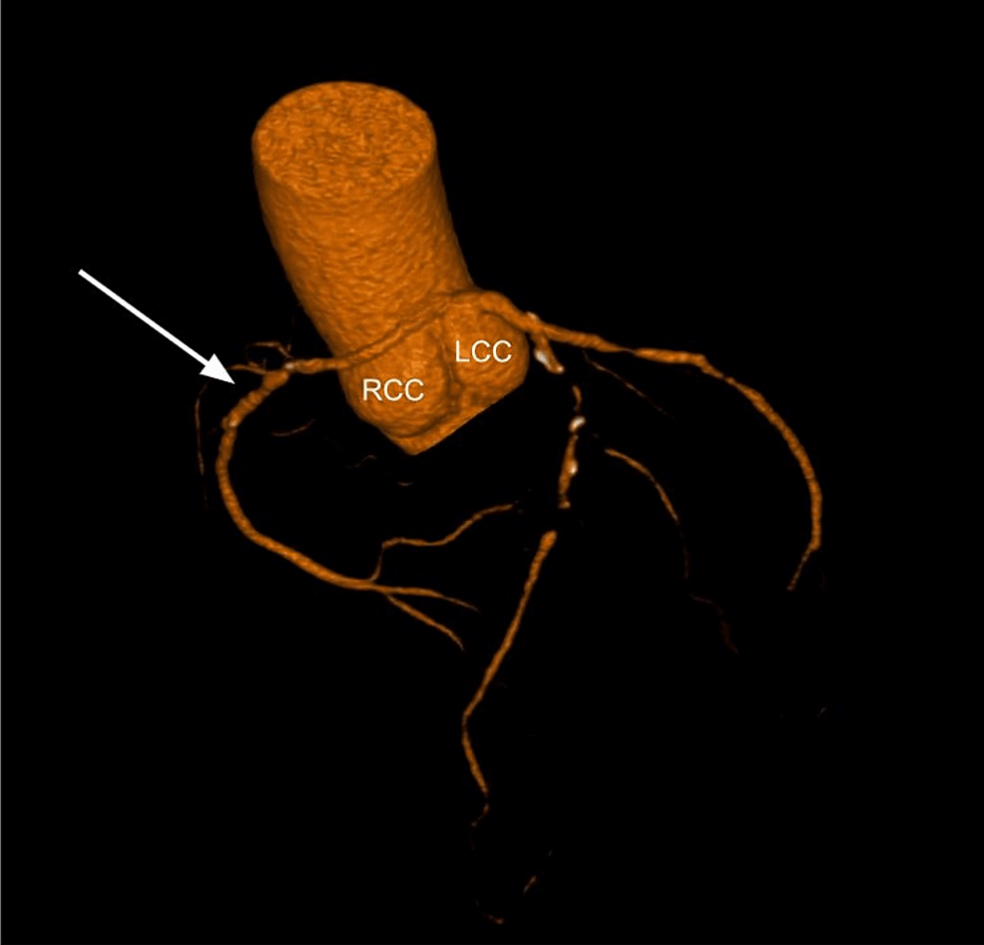
CTA coronary showing the anomalous origin of the RCA (arrow) arising from the left coronary cusp RCC: right coronary cusp; LCC: left coronary cusp; RCA: right coronary artery

A 62-year-old male with a past medical history significant for asthma and chest pain, with a workup in 2001 presumed to be non-cardiac in origin. He presented from a Micronesian Island for evaluation of chest discomfort. He reported that his chest discomfort has been intermittently occurring over the past year. He described it as a warm feeling on the left side of the chest, usually lasting for a few minutes, and mostly occurring when he is “sitting for too long.” He had a known chest pain history with a workup in 2001, including a stress echocardiogram and trans-thoracic echocardiography (TTE), which were unremarkable, and he was discharged with a diagnosis of bronchial asthma. His vital signs were a blood pressure of 112/90 mmHg, heart rate of 58 beats per minute, breathing 20 breaths per minute, and saturating 97% of ambient air. A cardiovascular exam showed no cardiac murmurs, rubs, clicks, or gallops. Labs are significant for a normal troponin generation 5 level of 7 ng/L (ref range <19 ng/L). Troponin remained negative at 7 ng/L on the serial draw. An electrocardiogram showed precordial ST changes consistent with a Brugada type I pattern (Figure 1). TTE was essentially unremarkable with a normal left ventricular systolic function and without regional wall abnormalities. Exercise stress testing revealed significant ST wave depressions on anterolateral and inferior leads with associated augmented vector right (AVR) elevation, which was concerning for multivessel disease and warrants further ischemic evaluation. The patient underwent left heart catheterization, which revealed right dominant circulation with three-vessel CAD, including mid-left anterior descending (LAD) artery chronic total occlusion (CTO) with the right to left collaterals, left circumflex, and RCA with the accompanied anomalous origin of RCA. Computed tomography angiography (CTA) coronary without calcium score (Figure 2) was ordered, which showed an anomalous origin of RCA from the left coronary cusp that has a malignant course between the aorta and pulmonary artery.

**Figure 3:**
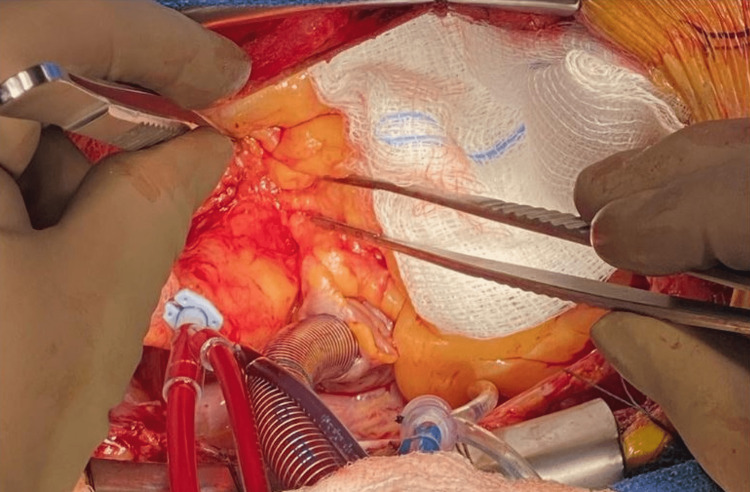
RCA after exiting the malignant course between the aorta and pulmonary artery RCA: right coronary artery

**Figure 4:**
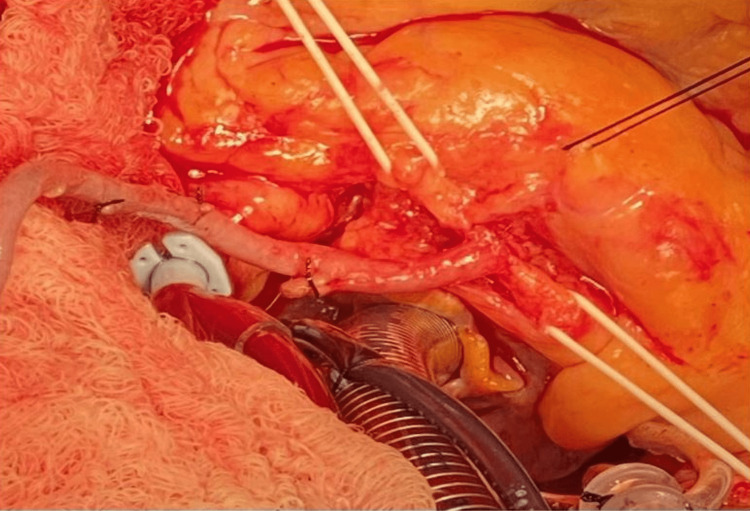
Close-up of vein anastomosis to the RCA RCA: right coronary artery

**Figure 5:**
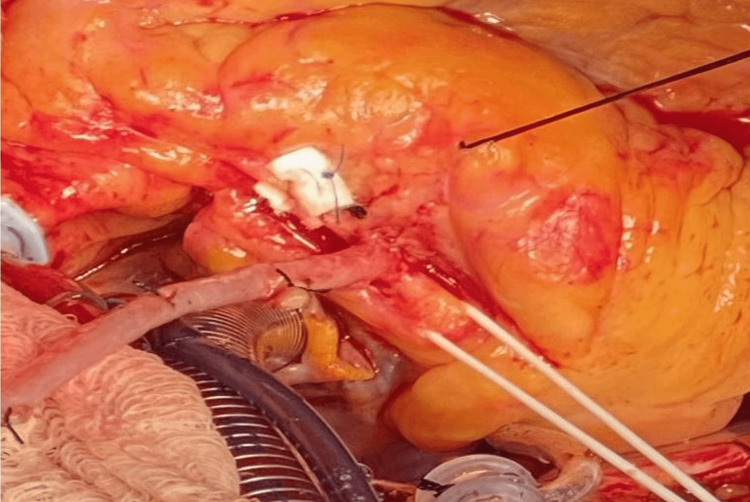
Close-up of pledgets ligating the proximal end of the RCA RCA: right coronary artery

The patient was referred to cardiothoracic surgery for consideration for a coronary artery bypass graft (CABG). The patient underwent CABG x4 including the left internal mammary artery to the left anterior descending artery, reversed saphenous vein graft to the right coronary artery, and a separate reversed saphenous vein graft to the obtuse marginal artery sequential to the diagonal artery. Ligation of the proximal RCA was also performed to prevent competitive flow under a resting state (see intraoperative pictures in Figures 3-5). His post-surgical course was unremarkable without significant complications. Aspirin desensitization was also conducted with no issues and the patient was started on daily aspirin.

## Discussion

Coronary artery anomalies are usually found incidentally during ischemic workups [5] but are important lesions that may have significant morbidity and mortality, which may present as chest pain, dyspnea, syncope, lightheadedness, cardiomyopathy, myocardial infarction, and even sudden cardiac death [4]. Pathophysiology is still poorly understood with numerous hypotheses that would explain the occurrence of cardiac deaths. It is postulated that the mechanism might be cumulative in nature causing patchy necrosis of the myocardium due to repeated ischemic events leading to malignant arrhythmias and sudden cardiac deaths [6]. Other theories are based on transient ischemia from kinking, compression, or thrombosis of CAAs [6-8]. Cautious identification of the patients who would benefit from pursuing diagnosis and treatment for CAAs is crucial, as surgical management can be equally dangerous if applied to the wrong set of the population. Younger patients of age 30 years and below diagnosed with isolated coronary artery anomalies are at increased risk of dying suddenly, despite exercising. A review of records in 1992 by Taylor et al. showed that 32% of 242 patients with isolated CAAs had sudden cardiac deaths, 45% of which were exercise-related [9]. High-risk features, such as the age of 30 years old and below, level of activities such as sports and general exercising, and specific anatomy such as anomalous right or left coronary artery arising from the contralateral coronary sinus would justify surgical correction [6,7,9,10]. According to the latest guidelines of the American College of Cardiology, coronary angiography, using catheterization, CT, or cardiac magnetic resonance imaging, is recommended for the evaluation of CAAs [11]. In this case, the patient underwent cardiac catheterization due to a positive stress test, which led to the incidental diagnosis of the anomalous origin of the RCA.

We believe that his presentation was consistent with his multi-vessel CAD leading to his chest discomfort given EKG findings on stress testing but was initially on a lower suspicion given the lack of cardiovascular risk factors and the non-exertional nature of his chest discomfort. In his case, it is also possible that his prior longstanding history of chest pain since 2001 might not be truly from bronchial asthma but due to the undiagnosed anomalous origin of his RCA. His Brugada type 1 pattern can be explained by his RCA occlusion causing Brugada phenocopy. Brugada phenocopy is distinct from true Brugada syndrome by the presence of reversible conditions such as ischemia, metabolic conditions, and mechanical compression [12]. Patients with CAAs can be managed conservatively with caution regarding physical activity, especially in the older population. However, this case is unique due to the CTO of the LAD with the right to left collaterals, causing the RCA to supply a much larger heart segment than normal, which increases his risk for SCDs. Also, due to the inter-arterial course and right dominant circulation, which further increased his risk for SCDs, surgical correction was warranted [5] in addition to CABG for his multi-vessel CAD.

## Conclusions

This case study presents a rare cardiac vascular variation in relation to its clinically significant diagnosis based on clinical features, investigation, and surgical intervention. It increases the knowledge of clinicians and educates them to prevent detrimental iatrogenic effects. The diagnosis of CAAs can be challenging and prone to misdiagnosis and maltreatment. Incidence may be higher than previously thought and may be beneficial to pursue in younger patients with ischemia-like symptoms. Further studies should be performed to identify the true incidence and high-risk features to guide medical practitioners regarding the risks, costs, and benefits of diagnosing and treating CAAs in the general population.
